# Ultrasonographic Assessment of Calcium Pyrophosphate Deposition Disease: A Comprehensive Review

**DOI:** 10.3390/jpm15070280

**Published:** 2025-07-01

**Authors:** Lissiane Karine Noronha Guedes, Letícia Queiroga de Figueiredo, Fernanda Oliveira de Andrade Lopes, Luis Fernando Fernandes Ferrari, Karina Rossi Bonfiglioli

**Affiliations:** Rheumatology Division, Hospital das Clinicas da Faculdade de Medicina de Sao Paulo, São Paulo 05403-010, Brazil; issiane.guedes@hc.fm.usp.br (L.K.N.G.); l.figueiredo@hc.fm.usp.br (L.Q.d.F.); fernanda.oalopes@hc.fm.usp.br (F.O.d.A.L.); luis.ferrari@hc.fm.usp.br (L.F.F.F.)

**Keywords:** calcium pyrophosphate deposition disease, CPPD, ultrasonography, chondrocalcinosis

## Abstract

Calcium pyrophosphate deposition disease (CPPD) is a common crystal arthropathy characterized by the deposition of calcium pyrophosphate crystals in joints and soft tissues. Ultrasonography (US) has emerged as a valuable imaging modality for diagnosing CPPD, offering real-time visualization of crystal deposits and joint inflammation. In the context of personalized medicine, US plays a critical role in enabling individualized patient assessment, facilitating early and accurate diagnosis, and supporting tailored therapeutic decisions based on specific imaging findings. This article reviews the ultrasonographic features of CPPD, their diagnostic utility, and clinical applications, emphasizing the relevance of US in stratifying patients and guiding personalized management approaches.

## 1. Introduction

Calcium pyrophosphate deposition (CPPD) disease arises from an inflammatory response to the pathological presence of calcium pyrophosphate crystals within the joints [1]. Its true prevalence remains unclear [2], and its clinical presentation is highly variable, sometimes mimicking other rheumatic disorders and ranging from asymptomatic cases to chronic inflammatory arthritis [2]. Risk factors for CPPD include advanced age and concomitant osteoarthritis [1].

Diagnosis is based on a combination of clinical findings, the identification of calcium pyrophosphate crystals under polarized light microscopy (when feasible), and characteristic imaging features. Given the limited accuracy and accessibility of synovial fluid analysis, imaging modalities offer a more practical and readily available alternative.

In this context, ultrasound (US) has gained significant interest as a diagnostic tool due to its widespread availability, safety, and real-time imaging capabilities. Its ability to enable dynamic bedside assessments of joints makes it a valuable modality in the evaluation of CPPD [1]. Furthermore, in the era of personalized medicine, ultrasonography holds promise not only for early and non-invasive diagnosis but also for tailoring disease management strategies based on individual imaging profiles. By identifying specific patterns of crystal deposition, US may help stratify patients according to disease severity and therapeutic response, contributing to more individualized and precise clinical decision-making.

## 2. Pathogenesis and Etiology

CPPD is characterized by pathologic mineralization that mainly occurs in the hyaline articular cartilage and fibrocartilage and occasionally extends to the synovial tissue, articular capsule, and tendon. The crystals are composed of calcium and inorganic pyrophosphate (PPi) [3,4]. In a physiological state, calcification of the cartilage matrix occurs during endochondral ossification of long bones in the embryonic phase and during callus formation after a fracture. This deposition is regulated by phosphate (Pi) and the PPi ratio in the cartilage matrix. Pi and PPi favor the formation and deposition of basic calcium phosphate (BCP) and calcium pyrophosphate (CPP), respectively. PPi’s role is not completely understood; nevertheless, it seems to inhibit BCP formation through Pi metabolization, thereby preventing cartilage calcification. However, if the PPi:Pi ratio increases, it promotes the pathologic deposition of CPP [3,5].

CPPD involves increased CPPD crystal formation, which is triggered by factors such as altered PPi production, genetic predisposition, aging, comorbidities, and chondrocyte abnormalities [3,6]. Substances like retinoic acid, ascorbate, thyroid hormones, nitric oxide, and PTHrp have been shown to stimulate crystal formation in in vitro studies [3,7]. Genetic factors, such as Nucleoside Triphosphate Pyrophosphohydrolase (NTPPPH) or ANKH hyperfunction, are implicated in CPPD pathogenesis. Comorbidities like hypophosphatasia, hypomagnesemia, hemochromatosis, and hyperparathyroidism further elevate this risk [3,6,8]. Chondrocytes in CPPD resemble those involved in hypertrophy and the mineralization of growth plate cartilage [3].

After calcium pyrophosphate crystals are formed, they can activate monocytes, macrophages, and endothelial cells, leading to inflammation. This can occur through three main mechanisms: recognition of the crystals by Toll-like receptors (TLR) in chondrocytes, opsonization of the crystals, and intracellular signaling through tyrosine kinases. These mechanisms ultimately induce the release of interleukin 1 (IL-1B), which perpetuates inflammation [6,8].

## 3. Clinical Aspects

CCP deposition, a condition known as chondrocalcinosis, can occur asymptomatically and is often detected on radiographs or ultrasonography. In contrast, CPPD (calcium pyrophosphate dihydrate deposition) disease involves symptomatic crystal deposition leading to acute and chronic arthritis. This can manifest in several phenotypes, resembling osteoarthritis, gout, or rheumatoid arthritis. Less commonly, CPPD can also present axial involvement, known as “crowned dens syndrome”, which is characterized by calcification around the odontoid process, resulting in neck pain and stiffness [3,6,9,10].

Acute arthritis, also called “pseudogout”, is characterized by the acute onset of mono- or oligoarthritis, occasionally accompanied by systemic manifestations such as fever and elevated acute phase proteins, resembling acute gouty arthritis. However, studies have shown that CPPD patients tend to be mostly female, older, and/or with a lower body mass index. Additionally, involvement of the knees is more frequent in CPPD acute arthritis, while the ankle and foot joints are more frequently affected in gout arthritis [10,11].

Chronic arthritis due to CPPD is typically nonerosive and polyarticular. CPPD can also lead to secondary osteoarthritis which, unlike primary osteoarthritis, may involve atypical sites such as the metacarpophalangeal joints and shoulders. A less common presentation is CPPD-related arthropathy, which mimics rheumatoid arthritis and is characterized by persistent inflammatory arthritis affecting both large and small joints, often in an asymmetric pattern.

## 4. Classification Criteria (ACR/EULAR 2023)

Prior to 2023, the only available diagnostic criteria for CPPD were the presence of CPP crystals in synovial fluid and chondrocalcinosis on conventional radiography, without considering the heterogeneity of clinical presentations or the role of advanced imaging techniques [12]. This resulted in significant diagnostic limitations, largely due to the low sensitivity of conventional radiography (CR) [13], limited access to modalities like ultrasound and dual-energy CT (DECT), and the high false-negative rate and inter-observer variability of synovial fluid analysis [14,15].

Filippou et al. (2016) [15] demonstrated that ultrasound outperforms radiographs in sensitivity, particularly in early or subtle disease presentations, by directly identifying crystal aggregates within cartilage and soft tissues. In contrast, conventional radiography, while widely accessible and specific for chondrocalcinosis, lacks sensitivity and may miss early or non-calcified lesions. Dual-energy computed tomography (DECT), although promising for crystal characterization, is less established for CPPD and currently limited by availability and cost.

The recent 2023 ACR/EULAR classification criteria [16] further underscore ultrasound’s role as a pivotal diagnostic tool alongside synovial fluid analysis, advocating for its integration into standard clinical assessment due to its non-invasive nature and superior diagnostic accuracy relative to conventional imaging. Together, these findings highlight ultrasound as a critical modality in the comprehensive evaluation of CPPD, complementing other imaging techniques and laboratory analyses. In alignment with the principles of personalized medicine, these criteria allow for a more nuanced diagnosis, recognizing that disease expression varies across individuals. The inclusion of ultrasound findings—such as calcifications within hyaline cartilage and fibrocartilage—as scoring elements (up to 48 points) reflects a shift toward incorporating patient-specific imaging data into diagnostic frameworks, enabling more tailored clinical management (Table 1).

## 5. Sonographic Findings of CPPD

**Figure 1:**
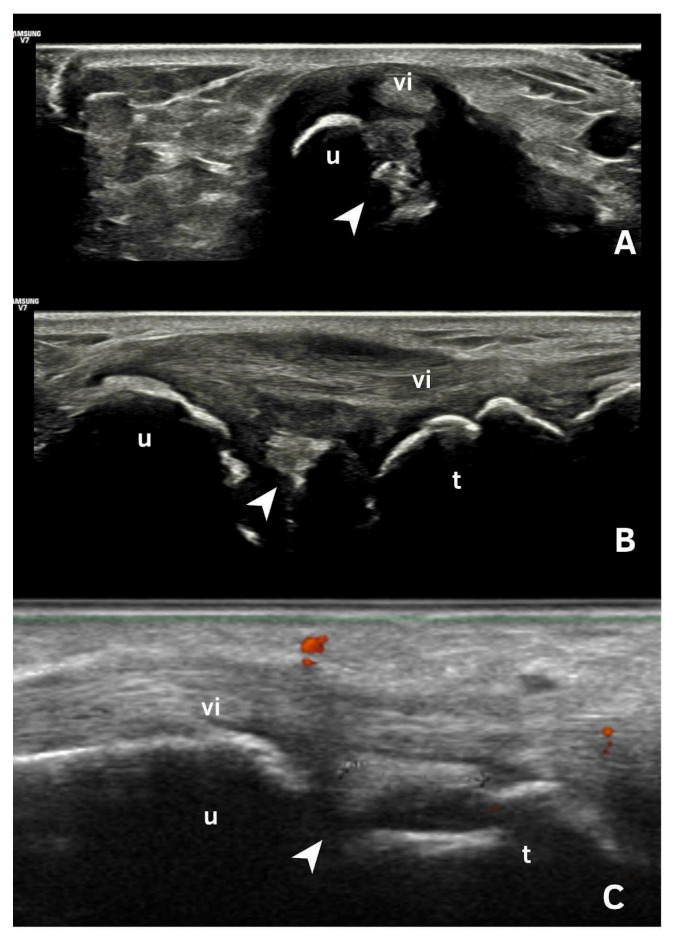
Ultrasonography of the triangular fibrocartilage of the wrist. (**A**): A transverse scan of the ulna showing CPP deposited in triangular fibrocartilage (arrow) beneath the tendon of the 6th extensor compartment of the wrist. (**B**,**C**): Longitudinal scan of triangular fibrocartilage (arrow) showing CPP deposits, beneath the tendon of the 6th extensor compartment of the wrist.vi: sixth extensor compartment, u: ulna, t: triquetrum.

**Figure 2:**
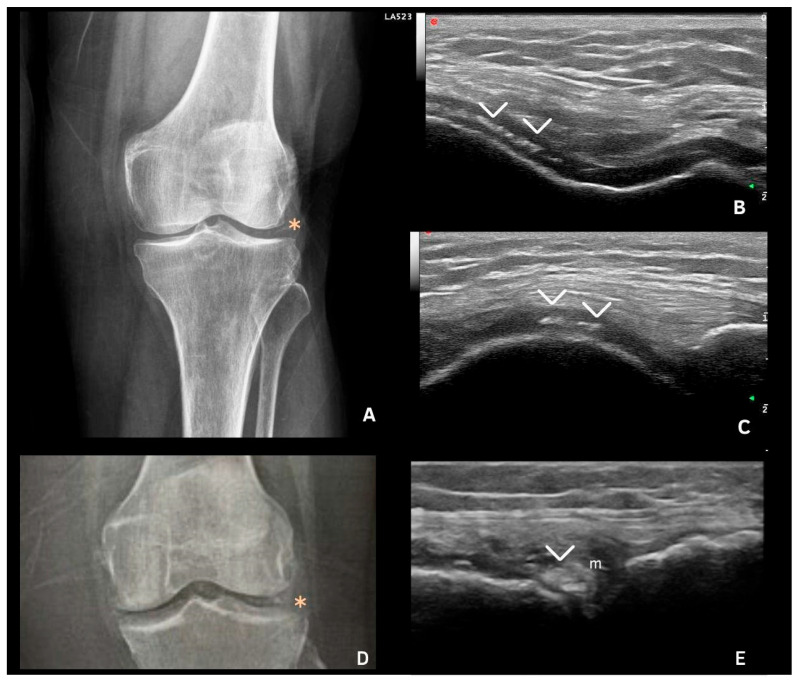
Imaging examples of calcium pyrophosphate (CPP) crystal deposition. (**A**) Knee radiograph showing linear calcification within the joint space (*), suggestive of chondrocalcinosis. (**B**) Axial ultrasonography of the femoral intercondylar region revealing hyperechoic deposits within the hyaline cartilage, aligned parallel to the cartilage surface without posterior acoustic shadowing (>). (**C**) Longitudinal ultrasound scan of the femoral condyle showing CPP deposition (>). (**D**) Knee radiograph with joint space calcification (*), consistent with CPP crystal deposition. (**E**) Longitudinal and lateral ultrasound views of the knee demonstrating CPP deposits in the lateral meniscus (>); m = meniscus. Source: personal archive.

Chondrocalcinosis and other CPPD-related findings in ultrasonography (US) include the visualization of calcium pyrophosphate (CPP) crystals in fibrocartilage, hyaline cartilage, tendons, and synovial fluid or tissue at various anatomical sites. These patterns were first characterized by Frediani et al. [17], who categorized them into three distinct types based on deposition in hyaline and fibrocartilage (Table 2, Figure 1 and Figure 2).

The establishment of the OMERACT CPPD Ultrasound Subtask Force in 2017 marked an important step toward standardizing ultrasonographic assessment. For the first time, this group defined CPPD-specific US features based on shape, echogenicity, anatomical location, and dynamic scanning (Table 3) [18]. These standardized definitions support a more reliable identification of disease manifestations across clinical settings, stratifying patients by severity and anatomical involvement and ultimately guiding individualized diagnostic and therapeutic decisions.

The consistency of these definitions was assessed across several anatomical sites, showing strong agreement in the menisci and hyaline cartilage of the knee but weaker agreement in other areas, particularly in tendons and synovial fluid. This is likely due to the wide range of conditions that can mimic CPPD crystals, such as enthesophytes and gout deposits in tendons, as well as gas bubbles in synovial fluid [19].

## 6. Deposits in Fibrocartilage

Deposits in fibrocartilage are typically hyperechoic, with echogenicity similar to that of the bone cortex, and may vary in shape and size. They often appear as punctate or amorphous hyperechoic aggregates (Figure 1) and remain fixed, moving with the fibrocartilage during dynamic evaluation. These deposits can be identified in the triangular fibrocartilage of the wrist and the menisci of the knee (Figure 1 and Figure 2E) [18,19,20,21].

The prevalence of fibrocartilage deposits is higher in the knee [22,23,24] and lower in the wrist, where it has been described as 56.25%. The authors of [18,23] evaluated inter- and intra-observer reliability for the identification of CPPD deposits in many sites, demonstrating a high reliability for knee menisci. However, further evaluation in other locations, such as the triangular fibrocartilage of the wrist, is still required. This is likely due to local technical challenges when assessing this area of the wrist, a lower frequency of findings compared to the knee, for example, and other local abnormalities that might mimic the presence of CPPD [18].

## 7. Deposits in Hyaline Cartilage

**Figure 3:**
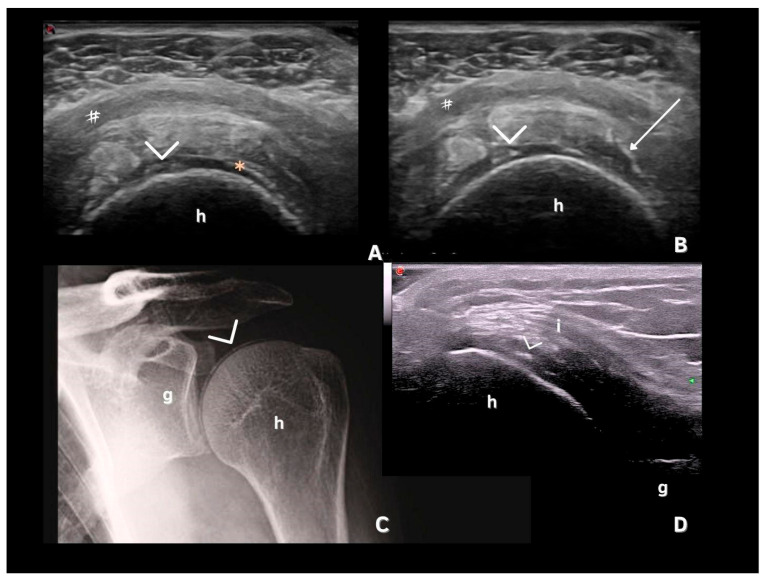
Images of CPP deposits. (**A**,**B**)—Transverse scan of the shoulder showing intra-cartilaginous calcifications (arrowhead); a pseudo-double contour sign (*); subacromial–subdeltoid bursitis (#); chronic supraspinatus tendinopathy with scattered tendinous calcifications (arrow). (**C**)—Shoulder X-ray showing CPP over the head of the humerus (>). (**D**)—US of the posterior recess of the shoulder, demonstrating infraspinatus tendon (i), and CPP deposits in hyaline cartilage (>); h—head of the humerus; g—glenoid. Source: personal files.

**Figure 4:**
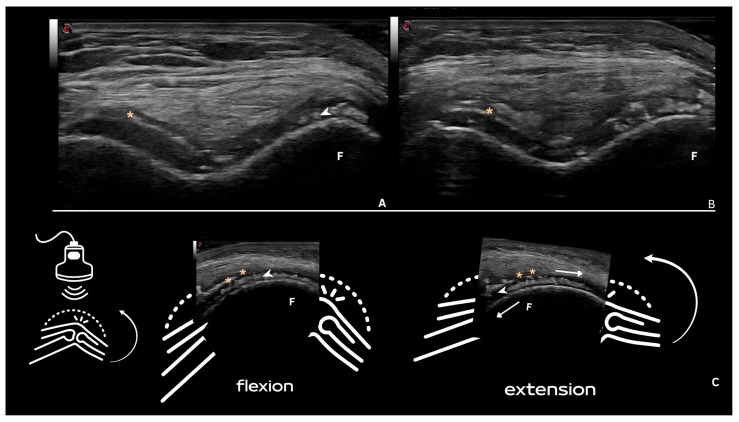
Pseudo-double contour. (**A**,**B**)—Femoral intercondylar transverse ultrasound scan with hyperechoic deposits within the hyaline cartilage parallel to the surface of the cartilage, without posterior shadowing (arrowhead). (*) Pseudo-double contour sign. F—Femoral condyle. (**C**)—Behavior during dynamic scanning (i.e., joint movement): The deposits within the cartilage remain fixed and move together with the femoral condyle (arrowhead), and the pseudo-double contour sign moves in the opposite direction (located at the chondro-synovial interface or in the joint capsule and adjacent ligaments. Source: personal files.

CPP deposits in hyaline cartilage appear as hyperechoic bands parallel to the cartilage surface, without posterior shadowing. Their size and shape vary with the disease stage, ranging from punctate spots (Figure 2 and Figure 3) to aggregates forming a pseudo-double contour (DC) (Figure 3A,B and Figure 4). Deposits forming a pseudo-DC are located at the chondro-synovial interface or within the joint capsule and adjacent ligaments. On dynamic scanning, intra-cartilaginous deposits remain fixed, moving with the cartilage and bone, whereas pseudo-DC deposits move in the opposite direction (Figure 4). In contrast, gout-related double contour signs move with the cartilage, as monosodium urate crystals deposit on its surface [20,21,22,25,26].

Filippucci et al. were the first to describe isolated hyaline cartilage involvement in crystal arthropathies [26]. Although theoretically present in any joint with hyaline cartilage, these deposits are most frequently observed in the femoral cartilage of the knee, with a prevalence of 28.1–30.7% [22,27]. While less common than fibrocartilage deposits and of lower sensitivity (59.5–68.7%), they demonstrate high specificity (96.4–97.6%) [26,28].

## 8. Deposits in Tendons

**Figure 5:**
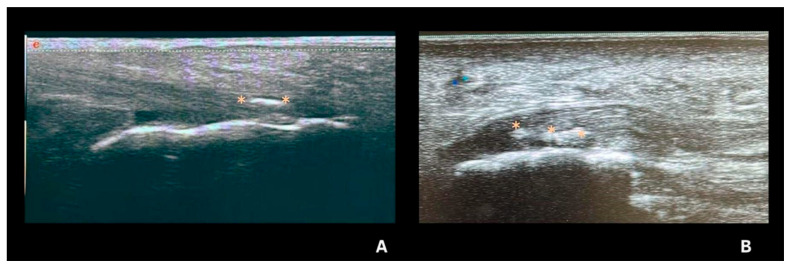
Tendon deposits in CPP. (**A**)—Achilles tendon with fine linear deposit that follows the direction of the fiber (orange asterisk *). (**B**)—Plantar fascia with deposits that follow the direction of the fiber and do not present acoustic shadow (orange asterisk *). Source: personal files.

CPP deposits in tendons appear as hyperechoic linear structures that may or may not produce posterior shadowing but remain highly echogenic even at low-gain settings and are unaffected by anisotropy. Distinguishing these from enthesophytes is important, as CPP deposits typically lack continuity with the bone cortex [18,19,20]. Tendon involvement in CPPD is less common, with the Achilles tendon and plantar fascia being the most specific sites—showing specificities of 100% and 96–100%, respectively. However, sensitivity is lower, at 57.9% for the Achilles tendon and 15.8% for the plantar fascia (Figure 5) [27,29], likely reflecting later involvement of these structures in CPPD progression [30].

Tendon deposits yielded low inter-observer agreement in a study by Filippou et al., which did not assess the Achilles tendon or plantar fascia, tendons with a high specificity for CPPD [18]. However, a high occurrence of tendon involvement with hyperechoic spots was observed in a study by Filippucci et al. [31] when evaluating the shoulders of patients with CPPD, with a prevalence of 45.6% for the supraspinatus, 21.7% for subscapularis, and 15.2% for the infraspinatus (Figure 3). The specificity and sensitivity were not evaluated, and these findings should ideally be considered together with other more specific sites, as they can be mistaken for other types of chronic tendinopathies that may occur concomitantly.

## 9. Deposits in Synovial Fluid or Tissue

**Figure 6:**
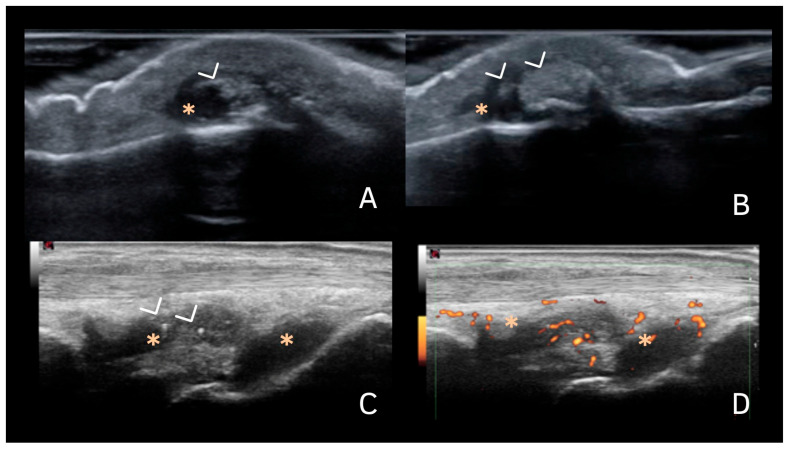
Synovial deposits of CPP. (**A**,**B**)—Longitudinal scan of distal interphalangeal joints, showing CPP deposits in synovial tissue; (*) synovitis; (arrowhead) CPP crystals. (**C**,**D**)—Longitudinal view of the wrist showing punctate hyperechoic synovial deposits and active synovitis with positive Power Doppler signal in a patient presenting with pseudogout phenotype. Source: personal files.

When deposits are observed in synovial fluid, they appear as hyperechoic spots of varying sizes, usually with well-defined margins and without posterior shadowing. They can be distinguished from synovial debris and proteinaceous material by their clear margins and the reflective properties of the crystals, which can be enhanced by adjusting the ultrasound settings to a low gain (Figure 6). These deposits are also mobile with joint movement and probe pressure [18,19,20,21]. Synovial fluid deposits also demonstrated low inter-observer agreement, likely due to a lack of standardized techniques for evaluating these sites [18].

## 10. Distribution of Deposits

According to two studies, the mean involvement of joints in patients with CPPD ranges from 2.9 to 4.7 joints per patient, with the knee being the most commonly affected site, followed by the wrist [11,32,33]. Other sites observed on US are the Achilles tendon, acromioclavicular joint, ankle and hip joints, pubic symphysis, and the metacarpophalangeal joints [11,32].

In the knee, CPP deposits identified in US imaging have shown high specificity and good sensitivity, mainly for the meniscal fibrocartilage. In previous studies, meniscal fibrocartilage calcification had a sensitivity of 53.3–90.5% and a specificity of 96.7–100% [28,34], and hyaline cartilage calcification had a sensitivity of 59.4–89% and a specificity of 91–100% [28,34,35]. Another study showed the best diagnostic value for the combined evaluation of deposits in the hyaline cartilage, menisci, and tendons altogether compared with only one or two of these or when compared with the evaluation of synovial fluid deposits [36]. Filippuci et al. [24] described the prevalence of some of the main pathologic US findings in CPPD, comparing them with findings in gouty arthropathy. They also found a higher prevalence of meniscal calcification (77.1%), followed by intra-cartilaginous hyperechoic spots on the hyaline cartilage of the femoral condyles (64.2%). Other types of involvement described were joint effusion, synovial hypertrophy, patellar and quadriceps tendon involvement, and popliteal cysts [24,37].

**Figure 7:**
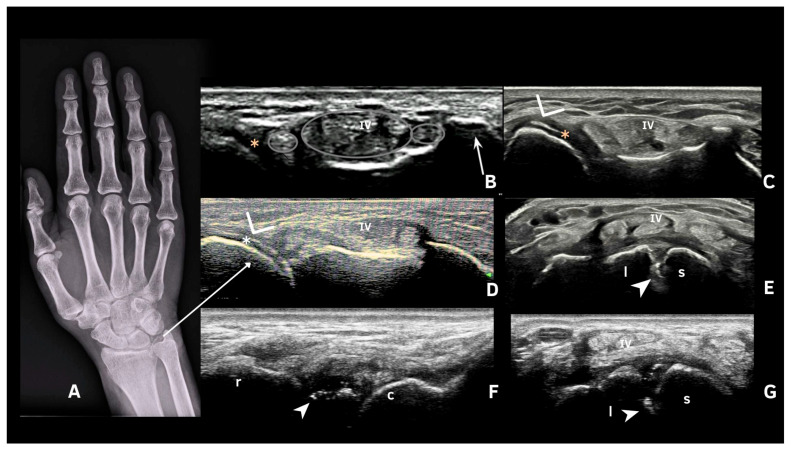
Wrist images. (**A**)—Radiograph showing CPP deposits in the distal radioulnar joint and triangular fibrocartilage (arrow). (**B**)—Transverse view at the level of the Lister’s tubercle of the radius (arrow) with the (iv) 4th extensor compartment. (*) Distal radioulnar joint. (**C**)—Linear deposition of calcium pyrophosphate in the distal radioulnar joint (*). (**D**)—Pseudo-double contour (*). (**E**)—Punctiform deposits (spots) in the scapho-lunate ligament (arrowhead); (l) lunate; (s) scaphoid. (**F**)—lLngitudinal scan of wrist scapho-lunate ligament with spots (arrowhead). (**G**)—Transverse scan of multiple spots on the scapho-lunate ligament (arrowhead). Source: personal files. c: capitate, IV: fourth compartment, l:lunate, r: radius, s: scaphoid.

The second most affected joint is the wrist, mainly due to calcification of the triangular fibrocartilage complex (TFC) but also with scapho-lunate ligament involvement [38]. The high prevalence of TFC US involvement, along with the considerable sensitivity (77.8–95%), specificity (85–90.6%), and accuracy (91%) found in previous studies, reinforces its importance for detecting CPPD on ultrasound [38,39,40]. However, in a minority of patients, it is difficult to obtain a good window of this spot, making it important to evaluate other sites at the wrist, such as the scapho-lunate ligament (Figure 7). A study by Cipolleta et al. [38] described this site as having high sensitivity and specificity and a greater accuracy than TFC involvement.

Filippucci et al. [31] described US findings in the shoulders of patients with microcrystalline diseases, including CPPD. The most frequent finding was acromioclavicular joint involvement (with osteophytes and calcifications in the fibrocartilage), followed by subdeltoid bursa effusion and supraspinatus tendinopathy [32]. These findings should be interpreted with caution, especially in older patients, who represent a population that is more likely to present with CPPD. Furthermore, the difficulty in adequately assessing the deep shoulder joint can affect the sensitivity and specificity of the US in assessing CPPD in this region. Di Matteo et al. (2019) described US CPPD findings in the hip, with a higher prevalence of deposits in the labrum fibrocartilage when compared with the hyaline cartilage [41].

Metacarpophalangeal joints may present with CPP deposits, with discrepant findings regarding its prevalence in CPPD patients reported in the literature, being described as 10% by Filippou et al. [32] and 40% by Cipolleta et al. [25], probably related to differences in patient selection between those two studies [25,32]. Diarthrodial joints usually present with four main types of deposits: within the hyaline cartilage (as hyperechoic spots), at the margin of the hyaline cartilage (as a DC sign), capsuloligamentous deposits, and other intra-articular deposits (e.g., within the fat pad and synovial proliferation). Such deposits have also been reported as intratendinous deposits in the flexor digitorum tendons [25]. Occasionally, when the disease manifests clinically as arthritis in the MCP joints, there may be accompanying inflammatory findings on ultrasound, such as synovial effusion, hypertrophy, and positive Power Doppler signal [25].

## 11. Ultrasound Technique for CPPD Evaluation

The ultrasound technique involves scanning specific joints to detect the characteristic signs of CPPD, such as hyperechoic bands or spots within the cartilage, as discussed above. With the advancement in high-frequency linear transducers (10–18 MHz), the wrists and knees—particularly the medial meniscus—are the most frequently assessed joints, showing a high prevalence of findings, similar to X-ray results [17,22,26]. In 2018, OMERACT US [18] established the characteristics and articulations to be included in CPPD evaluations. However, the definitions for CPPD have only been validated against histology in the knee [42].

**Figure 8:**
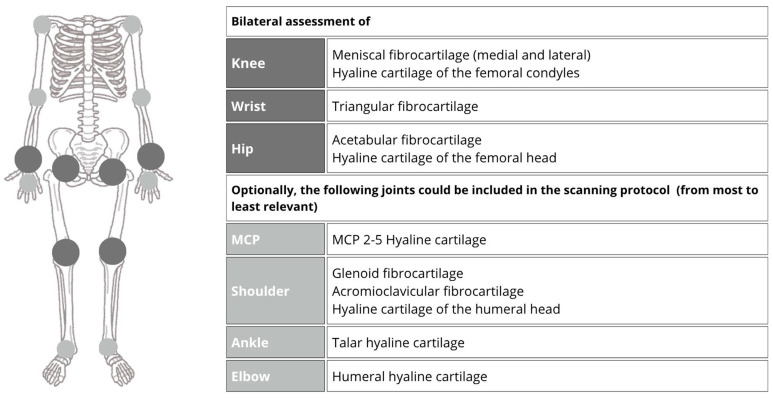
US scanning protocol for diagnosing CPPD disease. Adapted from Cipolletta et al. (illustration created by the authors, using graphic elements licensed for free use with no attribution required) [43].

Recent case series and practical observations have also revealed a high prevalence of these findings in the hip joints [13,33]. Because of these findings and the lack of concrete definitions for other joints proposed by OMERACT US, the authors of [43] aimed to determine the optimal US scanning protocol for diagnosing CPPD disease, conducting a cross-sectional study with 204 participants (half of whom had crystal-proven CPPD, and the other half were age- and sex-matched controls). A total of 20 joints, including the shoulders, elbows, wrists, metacarpophalangeal joints (second to fifth fingers), hips, knees, and ankles were assessed (Figure 8). These authors found that a reduced US scanning protocol involving the bilateral assessment of the knees, wrists, and hips showed excellent accuracy and feasibility for CPPD diagnosis. Specifically, detecting CPPD in two or more of these joints yielded a sensitivity of 96.7% and a specificity of 100%, with an average examination time of approximately 12.5 min. Evaluating other joints may be necessary in specific circumstances.

## 12. Limitations

Despite its advantages, musculoskeletal ultrasound has limitations in the evaluation of calcium pyrophosphate deposition disease (CPPD). Filippou et al. (2021) [42] highlighted that ultrasound sensitivity can be affected by the operator’s expertise and the selected scanning sites, potentially leading to underdiagnosis if the examination is incomplete. Cipolletta et al. (2024) [43] further emphasized the challenge of defining the optimal number and location of joints to scan for accurate CPPD detection, as crystal deposits may be patchy and unevenly distributed. Additionally, ultrasound may have limited ability to detect deep or small deposits compared to other imaging modalities, which can impact diagnostic accuracy.

## 13. Conclusions

Ultrasonography is a powerful tool for the evaluation of CPPD, offering detailed visualization of crystal deposits and inflammatory activity. Its non-invasive nature, real-time capability, and high sensitivity make it a useful modality in the diagnostic workup and management of CPPD, which is reflected in its inclusion in the latest ACR/EULAR classification criteria published in 2023. In the context of personalized medicine, ultrasonography allows for the individualized assessment of disease burden and joint involvement, supporting tailored diagnostic and therapeutic strategies. By enabling the dynamic monitoring of disease progression and response to treatment, US may play a key role in optimizing patient-specific care. This review contributes by integrating a visual approach aligned with the 2023 ACR/EULAR criteria, underscoring the practical implications of ultrasound as a frontline diagnostic tool in CPPD.

## Figures and Tables

**Table 1:** Image domain of ACR/EULAR classification criteria for CPPD disease.

**Hand or Wrist Osteoarthritis (Kellgren and Lawrence Score ≥ 2)**	
None or imaging not performed	0
Bilateral radiocarpal joint OA	+2
Two or more specific OA features (STTJ OA without first CMCJ OA, second/third MCPJ OA)	+7
**Imaging evidence of CPPD in symptomatic joint (US, CT, DECT, or X-ray)**	
None on any imaging	−4
None on X-ray (no other imaging performed)	0
Present on any imaging modality	+16
**Number of peripheral joints with CPPD on imaging**	
None	0
1 joint	+16
2–3 joints	+23
≥4 joints	+25

CMCJ: Carpometacarpal Joint; CT—computed tomography; DECT—dual-energy computed tomography; MCPJ—metacarpophalangeal joint; OA: osteoarthritis; STTJ—Small or Typical Target Joint(s); US—ultrasound.

**Table 2:** Patterns of calcific deposition (Adapted from Frediani et al. 2005 [17]).

1	Hyperechoic bands, parallel to the surface of the hyaline cartilage
2	Thin hyperechoic spots in fibrocartilage
3	Homogeneous hyperechoic nodular or oval deposits in fibrocartilage

**Table 3:** OMERACT definition for CPPD ultrasonographic findings.

Fibrocartilage	Shape: Deposits of variable shape. Echogenicity: Hyperechoic (similar to the bone cortex echogenicity). Localization: Within the fibrocartilage structure. Dynamic scanning: Remain fixed and move together with the fibrocartilage during dynamic assessment. Examples: Menisci, TFC, hip labrum, and acromioclavicular joint.
Hyaline cartilage	Shape: Deposits of variable shape. Echogenicity: Hyperechoic, without posterior shadowing. Localization: Localized within the hyaline cartilage. Dynamic scanning: The deposits remain fixed and move together with the hyaline cartilage. Examples: Knee and MCP joint.
Pseudo-double contour sign	Shape: Deposits of variable shape. Echogenicity: Hyperechoic, without posterior shadowing. Localization: Localized at the chondro-synovial interface or in the joint capsule and adjacent ligaments. Dynamic scanning: Moves in the opposite direction of cartilage and adjacent bone. Examples: Knee, proximal, and distal radio-ulnar joint.
Tendon	Shape: Multiple, linear (parallel to the tendon fibrillar structure and not in continuity with the bone profile). Echogenicity: Hyperechoic, generally without posterior shadowing, maintaining hyperechogenicity even at very low levels of gain, not affected by anisotropy as the surrounding tendon. Localization: Within the tendon. Dynamic scanning: Remain fixed and move together with the tendon.
Synovial fluid	Shape: Deposits of variable shape and size. Echogenicity: Hyperechoic (similar to the bone cortex echogenicity), generally without posterior shadowing Localization: Within the synovial fluid. Dynamic scanning: Mobile according to joint movement and probe pressure.

CPPD = calcium pyrophosphate deposition disease; TFC = triangular fibrocartilage complex; MCP = metacarpophalangeal. Adapted from Filippou et al. 2018 [18].

## Data Availability

Data sharing is not applicable.

## Abbreviations

The following abbreviations are used in this manuscript:

ACR	American College of Rheumatology
CMCJ	Carpometacarpal Joint
CPPD	Calcium Pyrophosphate Deposition Disease
CT	Computed Tomography
DECT	Dual-Energy Computed Tomography
EULAR	European Alliance of Associations for Rheumatology
MCP	Metacarpophalangeal
NTPPPH	Nucleoside Triphosphate Pyrophosphohydrolase
OMERACT	Outcome Measures in Rheumatology
PPi	Inorganic pyrophosphate
Pi	Phosphate
US	Ultrasonography
